# Successful Candidates in the New York State Licensing Examinations during May and June, 1903

**Published:** 1903-09

**Authors:** 


### Successful Candidates in the New York State Licensing Examinations during May and June, 1903.

[From the *New York Medical Journal,* August 15, 1903.]

### May Examination.

*State.*

Giuseppe Albano, 498 Chester Ave., Newark, N. J.; Florence E. Allen, 297 Alexander St., Rochester, N. Y.; J. Howard Allwein, 318 W. 46th St., New York; John C. Anderson, 46 E. 82d St., New York; William C. Armstrong, Windsor, N. Y.; David H. Atwater, 1109 E. Genesee St., Syracuse, N. Y.; John N. Bassin, 455 Henry St., Brooklyn, N. Y.; Clarence Beals, Salamanca, N. Y.; John L. Bishop, Whitesville, N. Y.; Fred. E. Bolt, Youngs, Delaware Co., N. Y.; Charles L. Bond, East Steamburg, N. Y.; Clarence H. Bonnell, 87 Madison Ave., New York; Donald Boyd, 152 Washington Ave., Albany, N. Y.; William S. Bryant, 33 E. 33d St., New York; Elliott Bush, Main St., Horseheads, N. Y.; John E. Canfield, Johnstown, N. Y.; Henry M. Chandler, South Orange, N. J.; Sylvester C. Clemans, 87 West St., Gloversville, N. Y.; Russell Clute, 70 Jay St., Albany, N. Y.; Henry J. C. Corrigan, St. Francis Hospital, 5th St. and Ave. B, New York; M. E. Costello, Willard, N. Y.; Patrick V. Costello, 214 Franklin St., New Haven, Conn.; Andrew J. Dolan, 1998 Lexington Ave., New York, care Dr. Upton; Archibald J. Douglass, Albany Hospital, Albany, N. Y.; Francis Benedict Doyle, 96 Boerum Pl., Brooklyn, N. Y.; Francis J. Doyle, 880 Madison St., Brooklyn, N. Y.; Clarence C. Duchscherer, 282 East St., Buffalo, N. Y.; Gaston H. Edwards, Kings Co. Hospital, Brooklyn, N. Y.; J. E. Eisenhart, 1195 Main St., Buffalo, N. Y.; George L. Fischer, 828 Jefferson St., Buffalo, N. Y.; David E. Fraser, Lyndonville, N. Y.; May Gibson, 10 Niagara Sq., Buffalo, N. Y.; Edward E. Gillick, 1508 Pine Ave., Niagara Falls, N. Y.; Marie M. Goldman, 11 W. 117th St., New York; Herman F. Graf, 282 Mulberry

St., Buffalo, N. Y.; Horace Greeley, 192 Warren St., Brooklyn, N. Y.; Dupree M. Hall, Westfield, N. Y.; Howard C. Hanscom, 142 W. 139th St., New York; James M. Happell, 609 Irving St., Olean, N. Y.; Albert J. Harris, 736-748 Jefferson St., German Hospital, Buffalo, N. Y.; Byron Haskin, Plessis, N. Y.; Henry Hirsch, 60 70th St., New York; Conrad R. Hoffman, Selkirk, N. Y.; R. Burdett Hoyt, Deposit, N. Y.; Harry Fisk Hall, Proctorsville, Vt.; Frank Jones, Himrod, N. Y.; Frank Keator, Accord, N. Y.; Augustine T. Kingston, Fordham Hospital, Fordham, New York; Leon M. Kysor, 9 Center St., Hornellsville, N. Y.; Orville N. Lewis, Ashland, N. Y.; Geo. G. Lindsay, 1738 Mousey Ave., Scranton, Pa.; Frederick J. MacDonald, St. Peter's Hospital, Albany, N. Y.; Miles A. McGrane, Watervliet, N. Y.; H. B. McIntyre, 203 Congress St., Brooklyn, N. Y.; J. A. McLeod, 26 Crescent Road, Toronto, Can.; D. S. McNaughton, 543 Bergen Ave., Jersey City, N. J.; Charles R. Marsh, 49 Chestnut St., Oneonta, N. Y.; Frank C. Maxon, Jr., Chatham, N. Y.; John C. Merchant, Nassau, N. Y.; Harry E. Mereness, Jr., 184 State St., Albany, N. Y.; John D. Moore, 151 Main St., Hartford, Conn.; J. Wesley Munro, 55 Wadsworth St., Buffalo, N. Y.; Louis I. Nisonoff, 228 Clinton St., New York; Thomas S. A. O'Connor, 787 4th Ave., Troy, N. Y.; Mark O'Meara, 123 No. Pearl St., Albany, N. Y.; A. M. Osness, 57 E. 115th St., New York; Albert W. Palmer, 287 South St., Lockport, N. Y.; Frederic J. Parmenter, 931 Prospect Ave., Buffalo, N. Y.; Louise Patterson, Amityville, N. Y., Long Island Home; George R. Pirie, 571 Lexington Ave., New York; Francis A. Prendergast, 340 Clinton St., Brooklyn, N. Y.; Fred. C. Purcell, 5 Allen St., Buffalo, N. Y.; Edwin D. Putnam, Smith's Mills, N. Y.; Maurice L. Radin, 309 Winslow Ave., Buffalo, N. Y.; Hyatt Regester, 61 Johnson Park, Buffalo, N. Y.; Edwin A. Reisenfeld, Buffalo German Hospital, Buffalo, N. Y.; Carroll J. Roberts, 56 Cottage St., Buffalo, N. Y.; N. L. Sapirstein, 29 W. 64th St., New York; Arthur J. Schneidenbach, 105 E. 75th St., New York; Sidney L. Scott, Bard Ave., West New Brighton, S. I., N. Y.; Virgil D. Selleck, 144 South St., Glens Falls, N. Y.; Millard F. Shafer, Cobleskill, N. Y.; E. F. Sibley, Albany City Hospital, Albany, N. Y.; Frank T. Smith, 14th and Jacob Sts., Troy, N. Y.; George H. Smith, 61 Hancock St., Little Falls, N. Y.; Jonathan M. Stafford, 229 Colchester Ave., Burlington, Vt.; Edward H. Storck, 225 E. Utica St., Buffalo, N. Y.; Max Sturm, care Dr. Henry Brooks, Post-Graduate Medical School and Hospital, New York; Chris. Lester Suess, West Main St., Lancaster, Erie Co., N. Y.; George C. Swerdfegger, 964 Jefferson St., Buffalo, N. Y.; John H. Telfair, Lying-in-Hospital, 18th St. and 2d Ave., New York; Frederick Tilney, 173 Amity St., Brooklyn, N. Y.; George W. Tong, 406 Monroe St., Brooklyn, N. Y.; Gennaro Tippolito, 9 Spring St., New York; James N. Vander Veer, 28 Eagle St., Albany, N. Y.; Isaac E. Van Hoesen, Coxsackie, Greene Co., N. Y.; Willard H. Veeder, Lyons, N. Y.; Michele Viggiani, 417 E. 12th St., New York; L. Edward Villiaume, 93 Watson St., Buffalo, N. Y.; Charles J. Walker, 602 Madison St., Brooklyn, N. Y.; William F. Walling, 176 Amity St., Brooklyn, N. Y.; George W. Warren, 2131 Broadway, New York; John L. Washburn, Sisters' Hospital, Buffalo, N. Y.; Harry M. Weed, Clyde, N. Y.; James H. Young, 138 W. Drinker St., Dunmore, Pa.

*Homeopathic.*

Thomas D. Blair, 306 E. 2d St., Plainfield, N. J.; Merritt G. Chambers, 86 Warren St., Glens Falls, N. Y.; Clarence W. Datesman, 87 Grove St., Passaic, N. J.; Frank P. Ekings, 197 Summer St., Paterson, N. J.; Judson K. Folwell, Port Washington, Long Island; James M. Gates, 391 Palisade Ave., West Hoboken, N. J.; Chalmers N. Kendrick, 17 Crescent Ave., Buffalo, N. Y.; Mary G. Potter, 39 West 60th St., New York; Bertha A. Rosenfeld, 162 West 99th St., New York; James E. Tytler, 113 W. 126th St., New York; James Walsh, 12 Union St., Cortland, N. Y.; Richard C. Warren, 217 Hazen St., Ithaca, N. Y.; Arthur C. Wilkes, Brewster, N. Y.

*Eclectic.*

Louis Cohen, 98 Allen St., New York; Cesar Deutsch, 64 St. Marks Pl., New York; Helen F. Gibson, 1841 Madison Ave., New York; Antonia J. Heffter, 399 Pleasant Ave., New York; Frederick Hollander, 239 E. 14th St., New York; Joseph Kallman, 64 E. 90th St., New York, care M. R. Arvine, M. D.; Germano Milite, Villa Ave., Bedford Park, N. Y.; Adelaide Mills, 310 Lexington Ave., New York; Herman Benjamin Schwartz, 980 2d Ave., New York; John W. Avery, Box 37, Noroton, Conn.; Harold S. Backus, Andover, Conn.; Lewis Bama, 108 E. 87th St., New York; Mabel H. F. Bancroft, West Philadelphia Hospital for Women, 4035 Parrish St., Philadelphia, Pa.; Ernest E. Banker, Fort Edward, N. Y.; Gioachino Barabini, 47 Catherine St., New York; Gennaro Sparano, 28 Mayfield St., Philadelphia, Pa.; Henrietta S. Tienken, 249 Ave. A, New York; Bertha A. Turkel, 92 E. 7th Ave., New York; Max H. Skou, 26 Charlton St., New York.

JUNE EXAMINATION.

*State.*

Samuel Abel, 71 E. 105th St., New York; I. Lathrop Allen, Jr., 358 Marion St., Brooklyn, N. Y.; Warren B. Andrews, Plantsville, Conn.; Walter H. Andrus, Children's Seashore House (Atlantic City, N. J.), Chelsea, N. J.; Martin Auspitz, 1517 2d Ave., New York; Howard A. Bayles, 262 Main St., New Rochelle, N. Y.; Allen Hoyt Beaman, Woodmere, Long Island, N. Y.; Erich C. Beck, 37 E. 31st St., New York; Chas. E. Bedell, 2 W. 116th St., New York; Frederick McK. Bell, Alms House Hospital, Blackwell's Island, New York; William S. Bellows, 27 Ackroyd Ave., Jamaica, N. Y.; Richard S. Benner, 214 Culver Road, Rochester, N. Y.; George H. Bentz, 309 E. 86th St., New York; Frederick S. Birchard, Birchardville, Susquehanna County, Pa.; Leonard Blumgart, 266 South Orange Ave., Newark, N. J.; Milton Bodenheimer, 336 E. 50th St., New York; Carl Boettiger, 390 Steinway Ave., Long Island City, N. Y.; John H. Borden, 52 W. 130th St., New York; Wm. E. Boyce, Park View Hotel, Van Cortlandt, N. Y.; J. Howard Branan, 133 2d St., Albany, N. Y.; Harry J. Brayton, St. Elizabeth's Hospital, Utica, N. Y.; Joachim Brennglass, 1478 1st Ave., New York; Abraham Brill, Manhattan State Hospital, Central Islip, L. I., N. Y.; Alfred J. Brown, Paul Smith's, N. Y.; Christopher W. Brown, 277 Division Ave., Brooklyn, N. Y.; Frank E. Brown, 591 Hancock St., Brooklyn, N. Y.; Alice G. Bugbee, 23 Hillside Ave., Waterbury, Conn.; Henry G. Bugbee, St. Luke's Hospital, New York; Jesse G. M. Bullowa, 46 E. 66th St., New York; Mortimer G. Burford, 83 S. 9th St., Brooklyn, N. Y.; Byron H. Caples, 527 W. 124th St., New York; Howard G. Case, 316 W. Onondaga St., Syracuse, N. Y.; Arthur F. Chace, South Swansea, Mass.; C. W. Chapin, City Hospital, New York; Wm. L. Clark, Box 302, Honesdale, Pa.; Gerhard H. Cocks, Post-Graduate Hospital, E. 20th St., New York; Frank Cohen, 116 E. 91st St., New York; Frank O. Cole, Lockport, N. Y.; Mary C. Connant, 201 Water St., Augusta, Me.; Albert H. Cook, 552 Spadina Ave., Toronto, Can.; Clarence C. Coryell, 108 Parker St., Ithaca, N. Y.; John F. Crawford, Surf Ave. and 21st St., Coney Island, Brooklyn, N. Y.; Archie I. Cullen, 1428 1st Ave., Watervliet, N. Y.; Henry M. Cullinan, 3744 Powelton Ave., Philadelphia, Pa.; Carlos E. Cummings, 560 Auburn Ave., Buffalo, N. Y.; C. E. Curtiss, Mexico, N. Y.; Frank H. David, 33 W. 126th St., New York; Walter W. Davis, 304 Stinard Ave., Syracuse, N. Y.; Ruth Demarest, State Hospital, Rochester, N. Y.; Oreste De Stefau, 739 S. 7th St., Philadelphia, Pa.; Paul Dolan, St. Vincent's Hospital, 11th St. and 7th Ave., New York; John F. Dooling, Passaic General Hospital, Passaic, N. J.; Sumner E. Douglass, Chateaugay, N. Y.; Gennaro Doyno, 303 E. 23d St., New York; Ed. H. Drozeski, 411 Myrtle St., Erie, Pa.; Francis E. Du Bois, Claverack, N. Y.; Roger Durham, M. E. Hospital, Brooklyn, N. Y.; Francis C. Edmonds,

Glen Cove, L. I., N. Y.; Chas. E. Elkins, Mexico, Oswego Co., N. Y.; Sigmund Epstein, 251 E. 52d St., New York; Frank Erdwurm, 108 Orient Ave., Jersey City, N. J.; James P. Erskine, 7 W. 15th St., New York Hospital, New York; Bryant Fassett, 1070 Madison Ave., New York; J. Wesley Faust, 129 Lexington Ave., New York; Lew H. Finch, Broadalbin, Fulton Co., N. Y.; Harry S. Fincke, 214 Grand Ave., Astoria, Long Island City, N. Y.; C. A. Finley, Post-Graduate Hospital, New York; Frederick A. Finn, 157 Danforth Ave., Jersey City, N. J.; H. S. Fish, Packer Hospital, Sayre, Pa.; Archie M. Fisher, Spencer, N. Y.; Eli Norman Foster, 243 Zara St., (Knoxville), Pittsburgh, Pa.; Pearl M. Foster, Whitesboro, N. Y.; Louis Franklin, 181 Bay View Ave., Jersey City, N. J.; Ernest V. Frederick, Cambellford, Ontario, Canada; Victor Frederickson, Bellevue Hospital, New York; G. A. Fried, 47 W. 87th St., New York; James L. Gallagher, 345 Eagle St., East Buffalo, N. Y.; Frank E. Gessner, 83 Burnet St., Newark, N. J.; Joseph H. Gettinger, 311 E. Broadway, New York; John E. Gleason, Oxford, N. Y.; F. W. Goddard, 4427 Osage Ave., Philadelphia, Pa.; Fred. Goldfrank, 12 E. 81st St., New York; Joseph E. V. Golding, 430½ Hart St., Brooklyn, N. Y.; Clinton E. Goodwin, 721 S. Crouse Ave., Syracuse, N. Y.; C. Sumner Gould, Walton, N. Y.; George C. Gould, 134 S. 6th Ave., Mt. Vernon, N. Y.; Herlwyn R. Green, 511 S. 42d St., Philadelphia, Pa.; Joseph Grief, 232 Rivington St., New York; Herman Gross, 135 Main St., Astoria, L. I.; Wm. P. Hall, Earlville, N. Y.; Samuel W. Hamilton, Randall's Island, New York; W. A. Hanor, Central Square, N. Y.; Glenn H. Hardy, Canisteo, N. Y.; George P. Harran, City Hospital, Newark, N. J.; Lasher Hart, 113 Elliot St., Syracuse, N. Y.; C. Morris Hatheway, 191 Prospect St., Willimantic, Conn.; Frank R. Haviland, Fulton, N. Y.; Royal S. Haynes, Presbyterian Hospital, 41 E. 70th St., New York; Max Hazay, 314 E. 3d St., New York; John A. Heitlinger, Stony Point, N. Y.; Walter H. Henning, 700 E. 185th St., New York; Louis Herlitzka, 245 E. 13th St., New York; Rudolph F. Herriman, Bushwick Hospital, Howard and Monroe Sts., Brooklyn, N. Y.; Julius J. Hertz, 302 E. 90th St., New York; Spencer L. Higgins, 403 Chestnut St., Roselle Park, N. J.; Anne A. Hintze, Women's Hospital, corner 22d and W. College Ave., Philadelphia, Pa.; Walter G. Hirseman, 408 Clinton St., Brooklyn, N. Y.; Horace P. Hoerle, Ridgewood, N. J.; Walter H. Holdrige, St. Francis Hospital, 5th St., between Aves. B and C, New York; Israel Horowitz, 243 E. 112th St., New York; Nathan M. Horowitz, 141 Monroe St., New York; Tasker Howard, 111 Mountain Ave., Montclair, N. J.; Rufus P. Hubbard, 116 W. 64th St., New York; Frederick B. Humphreys, 146 E. 37th St., New York; G. F. Inch, 297 Alexander St., Rochester, N. Y.; Peter Irving, 104 Madison Ave., New York; Maurice A. Jachnowitz, 403 E. 86th St., New York; Curtiss N. Jameson, 77 Jefferson Ave., Rochester, N. Y.; Frank A. Johnston, 197 W. Chestnut St., Kingston, N. Y.; Moses Kahn, 119 Grand St., Brooklyn, N. Y.; Louis Karmiohl, 90 Pitt St., New York; Howard T. Karsner, 66 Mt. Hermon Way, Ocean Grove, N. J.; J. A. Kearney, Archbald, Pa.; John J. Keating, 432 W. 160th St., New York; James M. Kent, 36 Church St., Putnam, Conn.; John D. Kernan, Jr., 307 W. 102d St., New York; Leo Kessel, 112 W. 72d St., New York; Chas. D. Kimball, Hecla, Westmoreland Co., Pa.; I. William Kingsbury, Presbyterian Hospital, New York; G. H. Kirby, Ward's Island, New York; John W. Kissane, Chateaugay, N. Y.; Charles W. Knauso, 92 First St., New York; Herbert W. Knight, 20 Genesee St., Binghamton, N. Y.; Joseph B. Knipe, 505 E. 83d St., New York; Wm. H. W. Knipe, 353 W. 24th St., New York; Louis M. Kommel, 87 Henry St., New York; H. A. Lakin, Frederick, Md.; John R. Le Comte, Lincoln Hospital, 141st St. and S. Boulevard, New York; Isaias Lehman, 1520 Washington Ave., New York; Horace L. Leiter, 137 W. 64th St., New York; Hugh H. Lenahan, 59½ Lansing St., Utica, N. Y.; Malcolm F. Lent, 1037 Howard St., Peekskill, N. Y.; Wm. Wolf Leseur, 127 E. 72d St., New York; Louis J. Levine, 202 E. 73d St., New York; Abraham A. Levy, Bedford San., Bedford Station, N. Y.; Jacob J. Levy, 717 E. Genesee St., Syracuse,

N. Y.; Louis F. Licht, 88 Cedar St., Brooklyn, N. Y.; W. C. Lippincott, 1707 Arch St., Philadelphia, Pa.; Thomas C. Lippman, 481 Amsterdam Ave., New York; John H. Long, Long Island College Hospital, Brooklyn, N. Y.; Henry D. Long, 401 S. Maple Ave., Greensburg, Pa.; Robert F. Ludwig, Breezehurst Terrace, Whitestone, L. I.; Marshall F. Lummis, Bridgeton, N. J.; W. J. Maby, 36 Main St., Cohoes, N. Y.; Donald G. McCaskey, 304 W. King St., Lancaster, Pa.; Wm. H. McCastline, 318 W. 57th St., New York; Samuel McClary, 3d, St. Timothy's Hospital, Roxborough, Philadelphia, Pa.; Wm. E. McCollom, St. John's Hospital, Atlantic and Albany Aves., Brooklyn, N. Y.; W. L. McFarland, Park Ave. Hotel, New York; John J. McGowan, 83 N. Fulton Ave., Mt. Vernon, N. Y.; Wm. H. McKinney, 4322 Manayunk Ave., Roxborough, Philadelphia, Pa.; Murdock D. Maclead, 228 E. 105th St., New York; John S. Macnie, 48 W. 83d St., New York; Wm. H. Magill, Bellevue Hospital, New York; Arthur R. Mandel, 150 E. 40th St., New York; Ralph K. Mead, Du Bois, Clearfield Co., Pa.; Avon A. Mendel, 1089 Lexington Ave., New York; Herman Mendlowitz, 36 Grand St., Brooklyn, N. Y.; Hermance Mosenthal, 181 W. 75th St., New York; Abraham Moskowitz, 83 Grand St., Brooklyn, N. Y.; James F. Nagle, Chicopee, Mass.; Thomas A. Neal, 131st St. and Amsterdam Ave., New York (J. H. Wright Hospital); Frederick L. Nelson, 212 E. 30th St., New York; N. W. Nelson, 1827 7th Ave., New York; Wm. H. Neville, 112 Oxford St., Syracuse, N. Y.; Leonard F. Nicoll, Newburgh, N. Y.; Frederick H. Nichols, 25 First St., Troy, N. Y.; Louis B. Nielson, St. Mary's Hospital, Philadelphia, Pa.; J. M. O'Neill, Massena, N. Y.; George G. Owens, Adams Centre, Jefferson Co., N. Y.; Joseph C. Palmer, 1000 E. Genesee St., Syracuse, N. Y.; Charles E. Panoff, 426 Stone Ave., Brooklyn, N. Y.; Alexander D. Parce, Springfield, Mo.; James McD. Parkinson, Manhattan State Hospital, E. 116th St., New York; Douglas C. Paterson, 105 W. 88th St., New York; George I. Pelgram, Benedicta, Maine; Max Perlman, 306 Delancey St., New York; Wm. Pfeiffer, 683 Greene Ave., Brooklyn, N. Y.; R. E. Pick, 234 E. 104th St., New York; H. Morton Pierson, Roselle, N. J.; Harry E. Plummer, 135 W. 104th St., New York; Ellen C. Potter, 2 William St., New London, Conn.; J. A. Pritchard, L. I. State Hospital, Flatbush, Brooklyn, N. Y.; G. A. Purpura, 218 Bruce St., Newark, N. J.; Camilla Quackenbush, Herkimer, N. Y.; James K. Quigley, Trumansburg, Tompkins Co., N. Y.; Selden I. Rainforth, Bayville, Long Island, N. Y.; S. J. Raphaelson, 92 East Broadway, New York; W. E. Recknagel, 164 W. 81st St., New York; Harvey L. Reese, 247 E. 21st St., New York; Ralph T. Richards, 963 Grigham St., Salt Lake City, Utah; Schuyler P. Richmond, 212 Park Ave., Syracuse, N. Y.; Charles F. Rissmeier, 2 Weiher Court, Bronx, New York; Hibbert R. Roberts, North Chili, N. Y.; Emanuel M. Robinson, 13 Orchard St., New York; Aaron Rokeach, 52 East 122d St., New York; Benjamin Romansky, 276 Madison St., New York; Morris Rosenbaum, 540 E. 5th St., New York; Herman Rosenberg, 10 W. 118th St., New York; N. Bertrand Ross, St. Mary's Hospital, Rochester, N. Y.; Valentine Ruch, Jr., Englewood, N. J.; Laurance P. Runyon, 14 Union St., New Brunswick, N. J.; Franz C. Ruppert, 171 E. 78th St., New York; Sebastian Saladino, 387 Broome St., New York; Laurel B. Sandall, 419 Lexington Ave., New York; S. J. Scadron, 11 Rutgers St., New York; Otto Scheina, 1997 Lexington Ave., New York; George A. Schnepel, 182 W. 82d St., New York; Morris Schoenfeld, 627 6th St., New York; Joseph Schrift, Workhouse Hospital, Blackwell's Island, New York; Herman Schwartz, 744 E. 5th St., New York; Samuel Schwartzman, 1289 Chesholm St., New York; Benjamin H. Searing, Bellevue Hospital, foot E. 26th St., New York, 2d div.; Keith Sears, Scarsburg, N. Y.; Jennie G. Seely, 437 Pennsylvania Ave., Waverly, N. Y.; Harlan Shoemaker, Municipal Hospital, Philadelphia, Pa.; John W. Short, 58 Liberty St., Camden, N. J.; S. Short, 222 E. 107th St., New York; Burton T. Simpson, 151 Front Ave., Buffalo, N. Y.; Joseph J. Sinnott, St. Vincent's Hospital, W. 12th St., New York; Penn Gaskill Skillern, Jr., 3400 Walnut St., Philadelphia, Pa.; De Verne C. Smith, Vernon Centre,

N. Y.; Everett G. Smith, 86 Maitland St., Toronto, Ont.; Frederick W. Smith, 721 Park Ave., Syracuse, N. Y.; Houghton C. Smith, Wilder, Vt.; Leroy James Smith, Bellevue Hospital, E. 26th St., New York; Louis Spanier, 290 Stanton St., New York; Elizabeth C. Spencer, Woman's Hospital, Philadelphia, Pa.; Edward W. Sprague, Garrett Hospital, Mt. Airy, Md.; Cynthia Steers, 19 Wendell Ave., Schenectady, N. Y.; Olga K. Steinbach, 1443 5th Ave., New York, care C. Bernard; Simon Steinbach, 1443 5th Ave., New York, care C. Bernard; George H. Stephens, 911 E. Genesee St., Syracuse, N. Y.; Adolph Stern, 1829 Croton Ave., Borough Bronx, New York; Alexander M. Stewart, Elmside P. O., Quebec, Canada; Isaac Stiefel, 158 Stanton St., New York; James W. Stiles, Jr., 710 Ocean Ave., Jersey City, N. J.; George Wm. Stimson, Neil House Block, Columbus, Ohio; Wm. C. Stolworthy, 238 13th St., South Brooklyn, N. Y.; F. D. Stone, Mexico, Oswego Co., N. Y.; Louis J. Stork, 703½ 3d Ave., Brooklyn, N. Y.; Katherine L. Storm, 1612 Diamond St., Philadelphia, Pa.; Abraham Strachstein, Beth Israel Hospital, Jefferson and Cherry Sts., New York; Gustave Strack, 215 E. 45th St., New York; Isaac Streep, 21 E. 117th St., New York; Edgar de M. Stryker, Raritan, N. J.; Harry R. Tarbox, Cooper Hospital, Camden, N. J.; Archibald W. Taves, 34 W. 85th St., New York; Richard A. Taylor, 215 W. 35th St., New York; Gustavo Testa, 267 N. 6th St., Brooklyn, N. Y.; Benjamin A. Thomas, Willow Grove Park, Montgomery Co., Pa.; Wellington A. Thompson, 265 Douglas St., Manchester, N. H.; Edward M. Tracy, 727 De Bevoise Ave., Astoria, L. I., N. Y.; Theodore Trumpp, 204 Nostrand Ave., Brooklyn, N. Y.; Isidore S. Tunick, 185 Allen St., New York; David C. Twichell, Saranac Lake, N. Y., care Dr. E. L. Trudeau; Jos. S. Van Dyke, Methodist Hospital, Philadelphia, Pa.; Le Roy P. Van Winkle, N. Y. Lying-in-Hospital, 2d Ave. and 18th St., New York; Harvey J. Vary, Skaneateles, N. Y.; H. W. Vickers, Canajoharie, N. Y.; Felix Villamil, 342 E. 30th St., New York; Emanuele Viola, 25 Roosevelt St., New York; Walter E. Vogt, 19 Belvidere St., Brooklyn, N. Y.; Otto Von Huffman, St. Luke's Hospital, New York; Edward W. Weber, 11 S. 5th Ave., Mt. Vernon, N. Y.; Arthur H. Weis, 17 State St., New York, care Mr. Aiton; Le Roy J. C. Wenger, 1040 N. 8th St., Reading, Pa.; Henry R. Weston, Felchville, Windsor Co., Vt.; Filip J. Wettervik, 224 E. 15th St., New York, care Dr. A. Vedin; Joseph N. Wickham, Corona, N. Y.; Karl D. Wood, State Custodial Asylum, Rome, N. Y.; Arthur R. Woods, 90 Main St., Nashua, N. H.; Floyd R. Wright, 202 E. State St., Ithaca, N. Y.; Joseph Witham Young, 127 W. 74th St., New York; Warren H. Young, 154 Claremont Ave., Montclair, N. J.; Eugene J. Zeiner, 706 Leonard St., Brooklyn, N. Y.; Hans Zinsser, 180 W. 59 St., New York; Morris Zucker, 358 E. 8th St., New York.

*Homeopathic.*

Renel A. Benson, Flower Hospital, 63d St. and Ave. A, New York; Cornelia C. Brant, 91 Macon St., Brooklyn, N. Y.; Edgar B. Cook, 100 Atkinson St., Rochester, N. Y.; Robert C. Fox, 192 Amity St., Flushing, N. Y.; Edwin P. Hall, Homeopathic Hospital, Albany, N. Y.; O. Du Bois Ingalls, Cumberland St. Hospital, Brooklyn, N. Y.; M. W. Johns, Norwich, N. Y.; F. Welles Kellogg, Box 976, Helena, Mont.; Walter E. Nichols, Henderson, Ky.; Mabelle J. Perry, 17 W. 101st St., New York; Daisy I. W. Rosenburg, 481 W. 159th St., New York; John E. Snodgrass, 224 Alexander St., Rochester, N. Y.; Thomas L. Thomson, 194 Main St., Torrington, Conn.; R. Percy Vivian, Barrie, Ontario, Can.; Lucy O. Wight, 438 W. 116th St., New York; George W. Whitney, 140 Fair St., Kingston, N. Y.

*Eclectic.*

Raffaele D'Angelo, 366 Broome St., New York; Sherman T. Davis, 13 E. 22d St., Chicago, Ill.; Jacob Haas, 1817 Madison Ave., New York; care M. Kraller; Solomon Ianovici, 5 1st Ave., New York; Luigi Zito, 181 Mulberry St., New York.

